# A c-di-GMP-Modulating Protein Regulates Swimming Motility of *Burkholderia cenocepacia* in Response to Arginine and Glutamate

**DOI:** 10.3389/fcimb.2018.00056

**Published:** 2018-02-28

**Authors:** Brijesh Kumar, John L. Sorensen, Silvia T. Cardona

**Affiliations:** ^1^Department of Microbiology, Faculty of Science, University of Manitoba, Winnipeg, MB, Canada; ^2^Department of Chemistry, Faculty of Science, University of Manitoba, Winnipeg, MB, Canada; ^3^Department of Medical Microbiology & Infectious Diseases, Max Rady College of Medicine, University of Manitoba, Winnipeg, MB, Canada

**Keywords:** cystic fibrosis, SCFM, *Burkholderia cenocepacia*, c-di-GMP, motility, BCAL1069, *cdpA*, *Burkholderia cepacia* complex

## Abstract

*Burkholderia cenocepacia* is an opportunistic bacterium that can thrive in different environments, including the amino acid-rich mucus of the cystic fibrosis (CF) lung. *B. cenocepacia* responds to the nutritional conditions that mimic the CF sputum by increasing flagellin expression and swimming motility. Individual amino acids also induce swimming but not flagellin expression. Here, we show that modulation of the second messenger cyclic dimeric guanosine monophosphate (c-di-GMP) levels by the PAS-containing c-di-GMP phosphodiesterase, BCAL1069 (CdpA), regulates the swimming motility of *B. cenocepacia* K56-2 in response to CF sputum nutritional conditions. Heterologous expression of WspR, a diguanylate cyclase, in *B. cenocepacia* K56-2 caused an increase in c-di-GMP levels and reduced swimming motility but did not affect flagellin expression or flagellar biosynthesis. After insertional mutagenesis of 12 putative genes encoding c-di-GMP metabolizing enzymes, one mutant of the locus BCAL1069 (*cdpA*), exhibited decreased swimming motility independent of flagellin expression in CF sputum nutritional conditions and an increase in intracellular c-di-GMP levels. The reduced swimming motility phenotype of the BCAL1069 mutant was observed in the presence of arginine and glutamate, but not of histidine, phenylalanine, or proline. The *B. cenocepacia* CdpA was also found to be involved in regulation of protease activity but not in biofilm formation. Altogether, these results highlight a role of *B. cenocepacia* BCAL1069 (CdpA) in sensing the nutritional conditions of the CF sputum and eliciting a pathogenic response that includes swimming motility toward amino acids and an increase in protease activity.

## Introduction

*C*yclic-*di*meric-*g*uanosine *m*ono*p*hosphate or c-di-GMP is an intracellular second messenger present in a wide range of bacterial species (Camilli and Bassler, 2006). In response to a change in the bacterial milieu, temporal, and spatial c-di-GMP levels are controlled through sensing and regulatory mechanisms, which alter bacterial physiology and phenotypes (Shanahan and Strobel, 2012). Initially associated with exopolysaccharide synthesis and biofilm formation, the role of c-di-GMP is now established in the transitioning of bacterial lifestyle from planktonic to sessile, cellular development, host cell adherence, virulence, and motility (Ross et al., 1987; Paul et al., 2004; Simm et al., 2004; Lee et al., 2010; Römling et al., 2013). C-di-GMP is synthesized and degraded by proteins containing GGDEF and EAL domains, respectively, named after the conserved signature motifs Gly-Gly-Asp-Glu-Phe (GGDEF) and Glu-Ala-Leu (EAL) (Galperin et al., 2001; Galperin, 2005). The GGDEF and EAL domains are typically linked to non-enzymatic domains that are involved in signal transduction systems (Galperin et al., 2001; Henry and Crosson, 2011). For instance, the GGDEF and EAL domains can be present in conjunction with signaling sensory domains such as GAF, PAS, Cache, HAMP, and a receiver domain, REC (Galperin, 2005; Römling et al., 2013). The sensory domains recognize small molecules and subsequently activate the receiver domain, resulting in functional changes in bacterial physiology and virulence factors, including motility (Galperin et al., 2001; Römling et al., 2013). A systematic study showed that three signaling domain-containing c-di-GMP proteins regulate swimming motility and pathogenicity in the rice pathogen, *Xanthomonas oryzae* (Wei et al., 2016). In *Salmonella Typhimurium*, the amino acid arginine was demonstrated to modulate c-di-GMP levels through a Cache-GGDEF domain-containing protein, leading to cellulose secretion and biofilm formation (Mills et al., 2015). These findings suggest that pathogenic bacteria can respond to environmental/nutritional cues and regulate virulence factors by modulating c-di-GMP levels through signal transduction pathways.

*Burkholderia cenocepacia* is a species of the *Burkholderia cepacia* complex (Bcc), a group of successful opportunistic bacteria with extremely versatile metabolism (De Smet et al., 2015; Eberl and Vandamme, 2016). Bcc species have been isolated from various ecological niches, such as soil, water bodies, plant rhizosphere, pharmaceutical products, and the lungs of people with the genetic disease cystic fibrosis (CF) (Mahenthiralingam et al., 2005). The diversity of Bcc ecological niches suggests that Bcc can sense different environmental settings and successfully elicit a response. For example, *B. cenocepacia* must possess mechanisms to sense the change from a relatively poor nutrient environment to the amino acid rich mucus present in the lungs of CF and trigger a response that results in successful colonization of the lung (Palmer et al., 2007a). Transcriptomic analysis of *B. cenocepacia* in nutritional conditions of CF sputum showed upregulation of flagellar biosynthesis genes, suggesting the importance of sensing the nutrients of the CF lung and the role of flagella in colonizing new environmental niches (Drevinek et al., 2008; Yoder-Himes et al., 2009, 2010).

We previously showed that synthetic CF sputum medium (SCFM) (Palmer et al., 2007a), induces motility of the clinical isolate *B. cenocepacia* K56-2 by upregulating flagellin synthesis and the number of flagella (Kumar and Cardona, 2016). SCFM mimics the nutrients of the CF sputum and is rich in amino acids. Interestingly, motility assays performed in a medium with individual amino acids also showed induced motility but the levels of flagellin were not affected (Kumar and Cardona, 2016). Considering the role of c-di-GMP in signal transduction pathways related to motility, we examined whether c-di-GMP metabolic genes regulate the motility of *B. cenocepacia* in response to amino acids. To inspect the role of c-di-GMP genes, we performed a systematic analysis of 12 sensory domain-coding c-di-GMP genes and assessed their role in swimming motility of *B. cenocepacia* K56-2. We found that a putative phosphodiesterase homologous to the *B. pseudomallei* CdpA (Lee et al., 2010), modulates intracellular c-di-GMP levels in response to amino acids and activates swimming motility. These findings deepen our understanding of the role of c-di-GMP signaling mechanisms in the adaptation of opportunistic pathogens to the host environment.

## Materials and methods

### Bacterial strains, plasmids, and growth conditions

The bacterial strains used in this study are summarized in Table 1. Primers are listed in Supplementary Table 1. The *B. cenocepacia* K56-2 wild type (WT) strain is a clinical isolate obtained from a CF patient and belongs to Rapid Amplification of DNA polymorphism (RAPD) and electrophoretic type (ET) 12. MOPS-glucose 20 mM and SCFM were prepared as described previously (Palmer et al., 2007a). The bacterial strains were grown in MOPS-glucose 20 mM or SCFM overnight, as indicated. Cells were washed in PBS and inoculated in SCFM, or MOPS-glucose 5 mM (glucose-minimal medium) with or without amino acids according to the experimental design. When appropriate, media was supplemented with trimethoprim (100 μg/ml for *B. cenocepacia* or 50 μg/ml for *Escherichia coli*), chloramphenicol (200 μg/ml for *B. cenocepacia* or 20 μg/ml for *E. coli*), kanamycin (25 μg/ml for *E. coli*), or rhamnose at 0.2% final concentration. To study the effect of amino acids, MOPS-glucose 5 mM was supplemented with individual amino acids at the same concentration present in SCFM.

**Table 1:** Strains and plasmids used in this study.

**Strains**	**Relevant genotype or phenotype**	**Reference or source**
***P. aeruginosa***		
PAO1	Wound isolate, laboratory strain	Holoway, 1955
PAO1/pSCrhaB2	Rhamnose inducible pSCrhaB2 plasmid in PAO1	This study
PAO1/ pWspR	Rhamnose inducible pWspR plasmid in PAO1	This study
***E. coli***		
DH5α	F- ϕ80 *lacZΔM15 endA1 recA1 hsdR17*(rK8 mK7) *supE44 thi-1 Δ gyrA96 (ΔlacZYAarg-F)U169 relA1*	Invitrogen
SY327	*araD* Δ(*lac pro*) *argE* (Am) *recA56* Rif^r^ *nalA λ pir*	Miller and Mekalanos, 1988
***B. cenocepacia***		
K56-2	Wild type (WT) K56-2 (LMG18863) strain, ET12 clonal related to J2315, cystic fibrosis isolate	Mahenthiralingam et al., 2000
WT/pSCrhaB2	Rhamnose inducible pSCrhaB2 plasmid in K56-2, Tp^R^	Cardona and Valvano, 2005
WT/pBKrhaB2	Rhamnose inducible pSCrhaB2 plasmid in K56-2, Cm^R^	This study
WT/pWspR	Rhamnose inducible pWspR plasmid in K56-2, Cm^R^	This study
***B. cenocepacia***		
**c-di-GMP mutants**		
WT::BCAM1160	K56-2, BCAM1160::pBCAM1160, Tp^R^	This study
WT::BCAM1554	K56-2, BCAM1554::pBCAM1554, Tp^R^	This study
WT::BCAM2836	K56-2, BCAM2836::pBCAM2836, Tp^R^	This study
WT::BCAL1020	K56-2, BCAL1020::pBCAL1020, Tp^R^	This study
WT::BCAM1161	K56-2, BCAM1161::pBCAM1161, Tp^R^	This study
WT::BCAM0580	K56-2, BCAM0580::pBCAM0580, Tp^R^	This study
WT:: BCAL1069	K56-2, BCAL1069::pBCAL1069, Tp^R^	This study
WT::BCAL2852	K56-2, BCAL2852::pBCAL2852, Tp^R^	This study
WT::BCAM0748	K56-2, BCAM07489::pBCAM0748, Tp^R^	This study
WT::BCAM2822	K56-2, BCAM2822::pBCAM2822, Tp^R^	This study
WT::BCAM2256	K56-2, BCAM2256::pBCAM2256, Tp^R^	This study
WT::BCAM1670	K56-2, BCAM1670::pBCAM1670, Tp^R^	This study
WT::BCAL1068	K56-2, BCAL1068::pBCAL1068, Tp^R^	This study
WT::BCAL1069/pBCAL1069	K56-2, BCAL1069::p1069, Tp^R^ pBCAL1069, Cm^R^	This study
**plasmids**		
pRK2013	Helper plasmid, RK2 derivative, Km^R^, mob^+^ tra^+^ ColE1	Figurski and Helinski, 1979
pGPΩTp	Ori_R6K_, Tp^R^ cassette, mob+, Tp^R^	Flannagan et al., 2007
pSCrhaB2	Rhamnose inducible promoter, ori_pBBR1_ rhaR, rhaS, P_rhaB_, Tp^R^, mob^+^	Cardona and Valvano, 2005
pKD3	Template plasmid for mutagenesis, Cm^R^	Datsenko and Wanner, 2000
pBKrhaB2	Rhamnose inducible promoter, ori_pBBR1_ rhaR, rhaS, P_rhaB_, Cm^R^ mob^+^	This study
pWspR	*wspR* of *P. aeruginosa* PA01 cloned in pBKrhaB2, Cm^R^	This study
pBCAM1160I	pGPΩTp, 326-bp internal fragment from BCAM1160	This study
pBCAM1554I	pGPΩTp, 493-bp internal fragment from BCAM1554	This study
pBCAM2836I	pGPΩTp, 320-bp internal fragment from BCAM2836	This study
pBCAL1020I	pGPΩTp, 360-bp internal fragment from BCAL1020	This study
pBCAM1161I	pGPΩTp, 377-bp internal fragment from BCAM1161	This study
pBCAL1069I	pGPΩTp, 380-bp internal fragment from BCAL1069	This study
pBCAM0580I	pGPΩTp, 345-bp internal fragment from BCAM0580	This study
pBCAL2852I	pGPΩTp, 338-bp internal fragment from BCAL2852	This study
pBCAM0748I	pGPΩTp, 317-bp internal fragment from BCAM0748	This study
pBCAM2822I	pGPΩTp, 302-bp internal fragment from BCAM2822	This study
pBCAM2256I	pGPΩTp, 324-bp internal fragment from BCAM2256	This study
pBCAM1670I	pGPΩTp, 324-bp internal fragment from BCAM1670	This study
pBCAL1068I	pGPΩTp, 343-bp internal fragment from BCAL1068	This study
pBCAL1069	pBKrhaB2, BCAL1069 gene	This study

*Km^r^, Kanamycin resistance; Tp^r^, Trimethoprim resistance; Cm^r^, Chloramphenicol*.

For genetic manipulations in *B*. *cenocepacia* K56-2 via triparental mating, the helper strain *E. coli* pRK2013 was used. *E. coli* SY327 Z-competent cells (Zymo Research, USA) were used to maintain the pBK1 plasmid. Polymerase chain reactions (PCR) were carried out with either *Taq* DNA polymerase (Qiagen) or HotStar HiFidelity *Taq* polymerase (Qiagen) with optimized conditions for each pair of primers. The DNA ligase and restriction enzymes (New England Biolabs) were used as recommended by the manufacturer. QIAquick purification kit (Qiagen) and QIAprep Miniprep kit (Qiagen) were used to purify PCR products and plasmids, respectively.

### Growth assays

Growth experiments were performed in 96-well format in a total volume of 200 μl. Bacterial cells were inoculated at a starting optical density at 600 nm (OD_600_) of 0.04 in triplicate. The 96-well microtiter plates were incubated at 37°C with continuous shaking for 24 h in a BioTek Synergy 2 plate reader. Readings were taken hourly at OD_600_ and values were converted to 1-cm-path-length OD_600_ by prior calibration with a GeneQuant™ III 4283, version 4283V1.6.

### Cloning of c-di-GMP genes in *B. cenocepacia*

To construct c-di-GMP modulating *B. cenocepacia* K56-2 (WT) strains, the *wspR* gene was PCR-amplified using *Pseudomonas aeruginosa* PAO1 genome as a template (primers described in Supplementary Table 1). The PCR amplicon and plasmid were digested using *Nde*I and *Xba*I, and the digested genes and plasmid were ligated using T4 ligase enzyme. This resulted in cloning of *wspR* gene into pSCrhaB2 under the rhamnose-inducible promoter. The resulting pWspR plasmid was introduced into the *B. cenocepacia* K56-2 WT strain, resulting in WT/pWspR.

### Insertional mutagenesis of c-di-GMP metabolizing genes

Insertional mutagenesis with the plasmid pGPΩTp was utilized (Flannagan et al., 2007). First, an internal fragment (~300–400 bp) of the gene of interest was PCR-amplified using forward and reverse primers (Supplementary Table 1). The PCR-amplified internal fragment and pGPΩTp were digested with the restriction enzymes, *Xba*I and *Eco*RI. The digested internal fragments and plasmid were ligated with T4 ligase enzyme, and the ligation products transformed into *E. coli* SY327. The resulting plasmids (Table 1) were introduced into *B. cenocepacia* K56-2 WT strain, generating exconjugants that were selected for trimethoprim resistance. Colony PCR was performed to confirm the insertion of the suicide plasmid in the target gene by using specific primers upstream to 5′ of the internal fragment and a primer that anneals with the plasmid (p53 reverse primer). The c-di-GMP mutants were named after the interrupted gene, for instance, WT::BCAL1069 mutant indicated insertional mutagenesis in the BCAL1069 gene.

### Complementation of the WT::BCAL1069 mutant

Plasmid pBKrhaB2 was created using pSCrhaB2 as the backbone. The dihydrofolate reductase gene of pSCrhaB2 was replaced with the chloramphenicol acetyltransferase (CAT) gene, which was amplified from pKD3 using primers, 876 and 877. The amplified CAT gene and pBKrhaB2 were digested with *Eco*RV and *Nsi*I. The digested CAT gene was ligated into digested pBKrhaB2 using T4 ligase enzyme. The resulting plasmid pBKrhaB2 was utilized for complementation experiments. To complement the WT::BCAL1069 mutant, the BCAL1069 gene was PCR-amplified using primers 851 and 852. The PCR-amplified BCAL1069 and pBKrhaB2 were digested with *Nde*I and *Xba*I, and the digested gene and plasmids were ligated using T4 ligase. The pBCAL1069 plasmid was introduced into the BCAL1069 mutant strain, creating the WT::BCAL1069/pBCAL1069 strain.

### Western blot

The WT and c-di-GMP mutant strains were grown overnight in SCFM. To ensure equal amounts of proteins were loaded, cell cultures were adjusted to an OD_600_ of 1.0 before taking equal volumes. To prepare the whole cell lysates, the cells were thawed, pelleted and resuspended in 2X SDS loading dye. After boiling, the whole cell lysates were separated by 12% SDS-PAGE to separate protein and transferred onto PVDF membrane. Then, the FliC protein production level was detected with the FliC specific primary polyclonal anti-flagellin antibody by incubating the membrane for 45 min at 4°C on the shaker. The polyclonal anti-flagellin antibody was raised against *B. pseudomallei* in rabbit (kindly gifted by Dr. David Speert). The membrane was then incubated with the secondary specific rabbit antibody tagged with alkaline phosphatase (Sigma-Aldrich, USA) for 45 min on a shaker. The ratio of primary and secondary antibodies dissolved in blocking buffer were 1:20,000 and 1:30,000, respectively. Finally, the FliC-primary antibody complex and secondary antibody interaction were detected with an NBT/BCIP detection kit (Roche, USA).

### Swimming motility assay

Overnight grown cell cultures were washed twice in PBS and the cell OD_600_ was adjusted to 1.0. To examine swimming motility of the WT and c-di-GMP mutants, 5 μl of bacterial inoculum was stabbed on 0.3% agar semi-solid plates. The plates, which were prepared on the same day, were then incubated statically at 37°C for 24 h. The motility halos were recorded quantitatively by measuring the circular zone of turbidity, which corresponds to the bacteria swimming away from the point of the inoculation. Motility halos of WT and mutants were compared and statistically analyzed. To examine mutants swimming motility, antibiotics were not added in the motility plates; however, rhamnose was added as inducer for gene expression.

### Electron microscopy

For electron microscopy sample preparation, a drop of the diluted overnight bacterial culture grown in SCFM was spotted on carbon-coated grids and stained with 2% uranyl acetate for 30 s. After drying the grids for 30 min, they were observed under a Hitachi H-7000 Transmission Electron Microscope at an operating voltage of 75 kV.

### Protease activity assay

Extracellular protease activity of WT and mutants were measured qualitatively using 1.5% agar containing 2% skim milk. The plates were inoculated with 5 μl of bacterial cell culture adjusted to an OD_600_ of 3.0 and incubated for 48 h at 37°C. Protease activity was indicated by a clear (lysis) zone around the bacterial colony. Protease activity of WT and mutants were compared and statistically analyzed.

### Biofilm formation assay

Bacterial attachment or biofilm formation were studied by measuring adherence to the wells of a 96-well polystyrene plastic plate using the method of Merritt et al., 2005. Overnight cultures were adjusted to initial inoculum of OD_600_ 0.04 in SCFM in the wells of the 96-well plate. The plate was then incubated statically at 37°C for 48 h. After the incubation period, planktonic bacteria were removed, and adherent bacteria washed three times in PBS, and then stained with 0.1% crystal violet for 30 min. The stain was removed carefully; the attached cells were again washed with PBS, and the stained bacteria eluted with 20:80 (v/v) acetone: ethanol mixture. Eluted CV stained bacteria readings were measured at OD_600_ using the BioTek plate reader.

### Extraction and quantification of c-di-GMP

The extraction of c-di-GMP in *B. cenocepacia* K56-2 was performed as previously described (Roy et al., 2013). Briefly, bacterial cell cultures were grown for 24 h in SCFM with or without supplementation with 0.2% rhamnose. A 20 milliliter volume of cell culture was standardized to OD_600_ 0.9, followed by washing the cells twice in ice cold H_2_0 at 16,000 × g for 3 min. Cells were resuspended in 1,000 μl of H_2_0 and boiled at 100°C for 10 min. To the same tube, 1,000 μl of ice cold 65% ethanol was added, and the suspension was vortexed for 15 s and centrifuged (16,000 × g, 3 min). The supernatant containing extracted c-di-GMP was transferred to a new microfuge tube, and the extraction procedure was repeated. The supernatant from the two extractions was dried using a vacuum centrifuge (Thermo, SpeedVac). The visible white pellet, nucleotide extract, was resuspended in 200 μl H_2_0, followed by filtering (0.2 um filter, GE). The nucleotide extract was stored at −80°C until used. Using reverse-phase high-performance liquid chromatography (RP-HPLC), c-di-GMP was detected and quantified by injecting 40 μl of nucleotide extract into the column.

Detection and quantification of intracellular c-di-GMP was performed using a Waters HPLC Separation Module 2695 equipped with an autosampler, degasser and UV/Vis detector set to 256 nm (PDA Detector Model 2996). Separation of molecules in the extract was achieved by μBondapak™ Waters C18 (3.9 × 300 mm) column particle diameter of 15–20 μm, with 125 Å pores at a flow rate of 1 ml min^−1^. Solvents containing methanol 100% (solvent A) and trifluoroacetic acid 0.075% (solvent B) were used. To elute c-di-GMP, the following gradient was used: 0.01–10 min, 5% solvent A (= 5% solvent A and 95% solvent B); 10–20 min, 5–20% solvent A; 20–25 min, 20% solvent A, 25–30 min, 20–5% solvent A. The retention time of standard c-di-GMP was observed ~7.6 min and the UV spectra 200–600 nm was used to confirm the identity of c-di-GMP. The area under the curve or peak was used to quantify the c-di-GMP concentration, and change in the c-di-GMP levels in strains are represented in percentage.

### Bioinformatics analysis

The amino acid sequence of c-di-GMP related proteins were retrieved from the Burkholderia.com and UniProt databases (Wu et al., 2006; Winsor et al., 2008). Domains in putative c-di-GMP metabolizing proteins were predicted using the SMART (Simple Modular Architecture Research Tool) program (Schultz et al., 2000; Letunic et al., 2015). To perform alignment of amino acid sequence, a multiple sequence alignment program, MAFFT, was used (Katoh and Standley, 2013). Further, the alignment viewer tool AliView was utilized to observe GGDEF and EAL motifs in the multiple aligned sequence (Larsson, 2014).

### Statistical analysis

An unpaired Student's *t*-test was used to analyze data from two groups, and one way ANOVA followed by the Dunnett's multiple comparisons test was used to analyze data for more than two groups. *P*-values were calculated using GraphPad Prism version 6 for Windows 7, GraphPad Software (La Jolla California USA). Differences were considered significant when the *P*-value was less than 0.01.

## Results

### Increased c-di-GMP levels regulate swimming motility of *B. cenocepacia* K56-2

**Figure 1:**
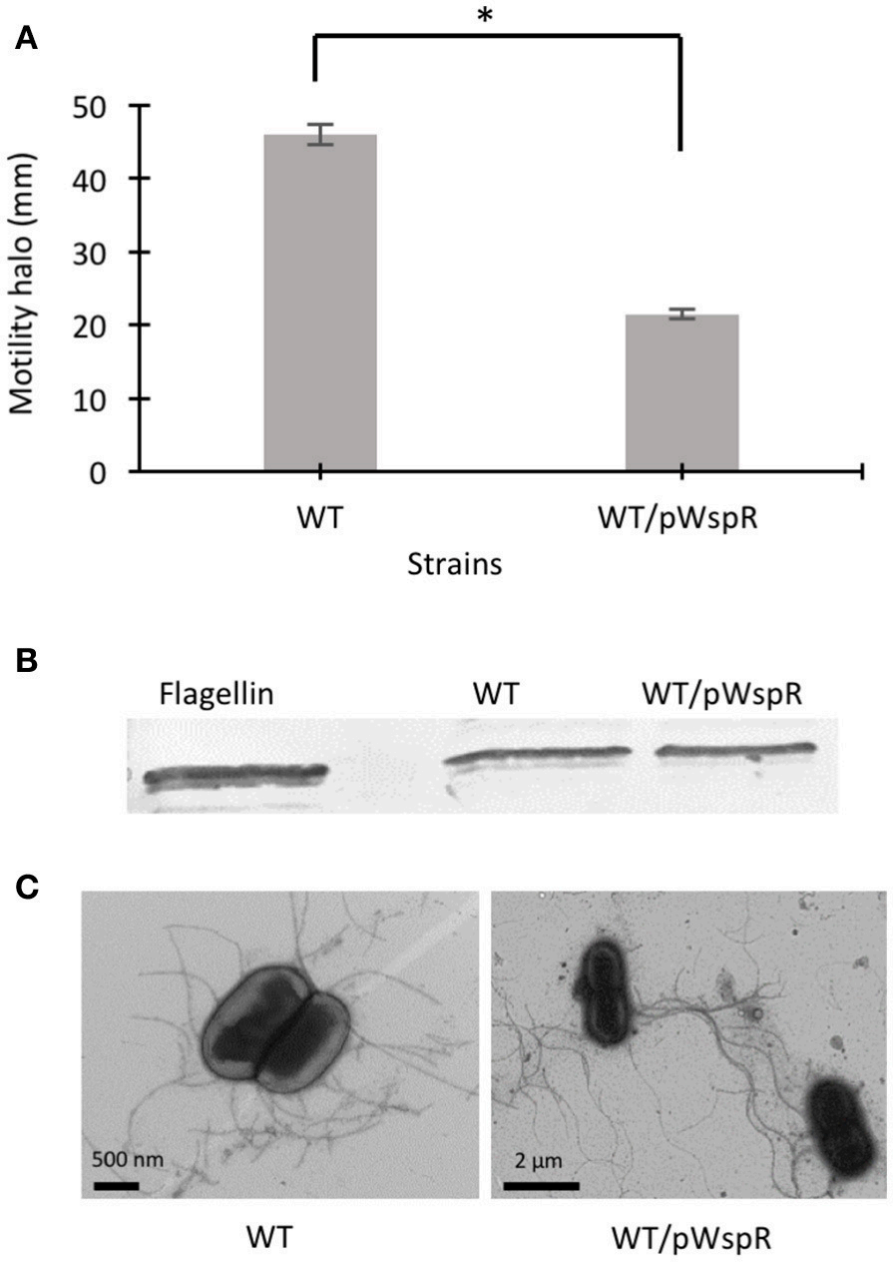
The effect of c-di-GMP modulating conditions on swimming motility, flagellin expression, and flagella biosynthesis of *B. cenocepacia* K56-2 WT. **(A)** Motility of the strains was examined in semi-solid SCFM 0.3% agar plates after 24 h at 37°C. Experiments were performed three-times independently in duplicate. ^*^Denotes significant *p*-values (*p* < 0.01). **(B)** Whole cell lysates were used to separate proteins by 12% SDS-PAGE followed by detection of flagellin protein using anti-flagellin primary antibody and alkaline phosphatase crosslinked to secondary antibody. From two Western blots, one representative experiment is shown. **(C)** Electron micrographs of uranyl acetate stained WT strains displaying flagella under c-di-GMP varying conditions.

Modulation of c-di-GMP levels can change flagellar gene expression (Hickman and Harwood, 2008) or affect flagellar rotation, resulting in altered motility (Boehm et al., 2010; Baraquet and Harwood, 2013). In a previous study, we showed that the CF sputum nutritional conditions increased the swimming motility of *B. cenocepacia* K56-2 through upregulated flagellin expression and a change in the flagellation pattern (Kumar and Cardona, 2016). However, in the presence of individual amino acids, the motility of the K56-2 strain was upregulated independent of flagellin expression, possibly through the mechanism of regulation of flagellar function. To address whether c-di-GMP regulates the swimming motility of *B. cenocepacia*, we cloned the *wspR* gene from *P. aeruginosa* PAO1 and overexpressed it in *B. cenocepacia* K56-2 (WT). The *wspR* gene encodes a well-characterized diguanylate cyclase enzyme that modulate intracellular c-di-GMP levels by synthesizing c-di-GMP (Hickman et al., 2005). Next, we evaluated the swimming phenotype of *B. cenocepacia* overexpressing WspR in SCFM, which mimics CF sputum nutritional conditions (Palmer et al., 2007b). Figure 1A shows that the swimming motility of the WT/pWspR strain is reduced under the WspR protein overexpression condition. Similarly, when WspR was overexpressed in *P. aeruginosa* PAO1, overexpression of WspR decreased swimming motility (Supplementary Figure 1).

To confirm that overexpression of the WspR protein changed the c-di-GMP levels in the WT/pWspR strain, we measured intracellular c-di-GMP levels in the WT/pWspR strain by high-pressure liquid chromatography (HPLC). The chromatogram of the WT/pWspR nucleotide extract shows an increased peak at the retention time of ~7.6 min, which corresponds to the standard control elution time, in comparison to the WT nucleotide extract (Supplementary Figure 2). The UV trace of the c-di-GMP standard corresponds to the peak of WT/pWspR (Supplementary Figures 2B,C). To confirm that the observed peak corresponded to c-di-GMP, we spiked the nucleotide extracts of strains with c-di-GMP. The same peak with a higher intensity was observed, confirming that the elution time and UV trace corresponded to c-di-GMP.

To investigate if induced intracellular c-di-GMP levels affect flagellar function in the WT strain at the post-translational level, we detected flagellin protein expression by Western blot and observed the presence of flagella by electron microscopy. The flagellin levels in the WT/pWspR strain were similar to the WT strain (Figure 1B, Supplementary Figure 1). The slight difference between the migration of the native flagellin and the recombinant protein control expressed in *E. coli* is likely due to the flagellin glycosylation of *B. cenocepacia* (Hanuszkiewicz et al., 2014). In agreement with the lack of upregulation of flagellar biosynthesis, the flagellation patterns of the WT and c-di-GMP modulating strain WT/pWspR was similar, as observed by electron microscopy (Figure 1C). Taken together, these results indicate that the induced c-di-GMP levels reduce the motility of *B. cenocepacia* K56-2 (WT) strain through a mechanism that is independent of flagellin expression levels or changes in the flagellation pattern.

### Mutational analysis of GGDEF/EAL-signaling-domain coding genes reveals that BCAL1069 plays a role in the swimming motility of *B. cenocepacia* K56-2

**Figure 2:**
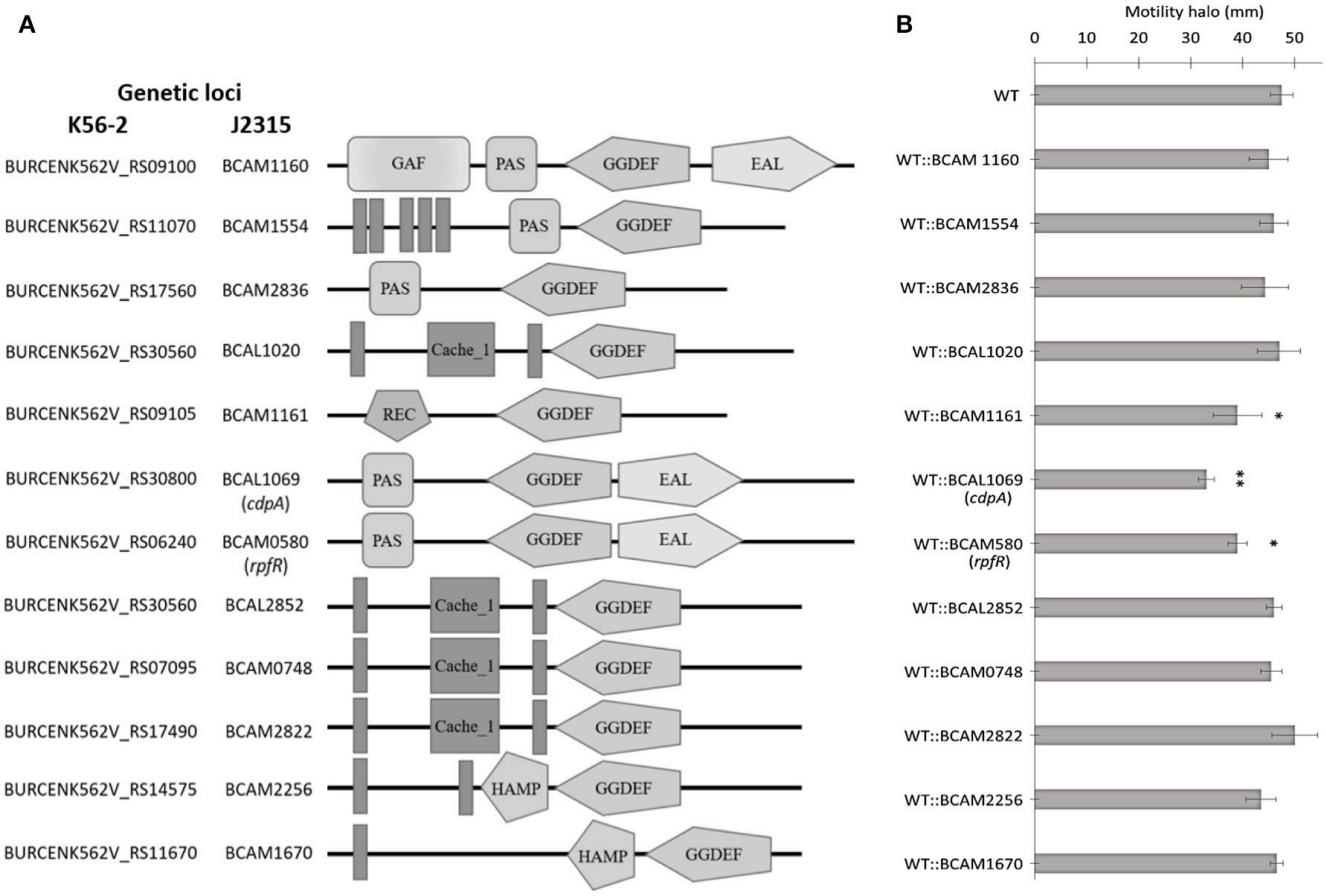
Predicted domains coded by c-di-GMP metabolic proteins and their role in swimming motility of *B. cenocepacia* K56-2 WT. **(A)** Putative proteins with c-di-GMP modulating and signal transduction domains as predicted by SMART. Amino acid sequences were obtained from the genome of *B. cenocepacia* J2315. EAL, putative diguanylate phosphodiesterase; GG(D/E)EF, putative diguanylate cyclase; PAS, *P*er/*A*RNT/*S*im; GAF, c*G*MP-specific phosphodiesterase/*A*denyly cyclase/*F*hlA; REC, cheY-homologous receiver domain; Cache, *CA*lcium channels and *CHE*motaxis receptors; HAMP, *H*istidine kinases/*A*denylate cyclases/*M*ethyl accepting proteins/*P*hosphatases; Vertical bars, transmembrane domain. Domains are not drawn to scale. **(B)** The swimming motility of the mutants was examined in semi-solid SCFM 0.3% agar plates after 24 h at 37°C. Three independent experiments were performed in duplicates (^*^p < 0.01; ^**^p < 0.001).

**Figure 3:**
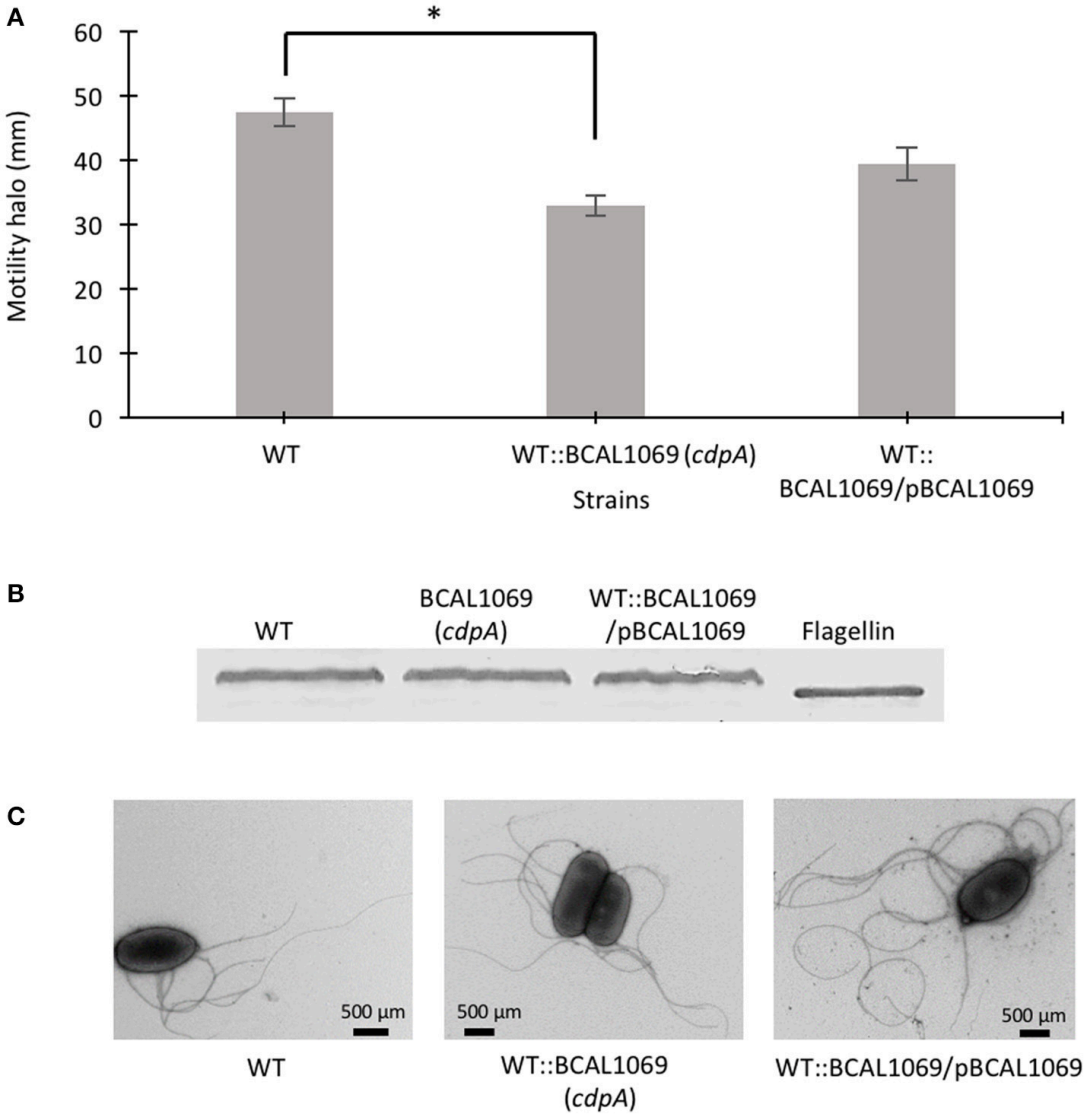
Role of BCAL1069 (*cdpA*) motility in swimming motility. **(A)** The swimming motility of the strains was examined in semi-solid SCFM 0.3% agar plates at 37°C after 24 h. The motility assay was performed three-times independently in duplicates (^*^p < 0.01). **(B)** To detect flagellin protein, proteins from whole cell lysates of strains were separated on 12% SDS-PAGE followed by flagellin detection using anti-flagellin primary antibody and alkaline phosphatase cross linked to secondary antibody. Western blots were performed twice. One representative experiment is shown. **(C)** The presence of flagella in the WT and mutant strains were observed using transmission electron microscope.

C-di-GMP metabolizing protein encoding genes are numerous, ranging from ~30 to 50 in pathogenic bacteria such as *E. coli, P. aeruginosa*, and *Vibrio cholerae* (Waters et al., 2008; Ha and O'Toole, 2015; Povolotsky and Hengge, 2015). Despite a plethora of c-di-GMP data on other pathogenic bacteria, our knowledge regarding the roles of putative c-di-GMP metabolic genes in adaptation to the host environment is limited in *B. cenocepacia*. We used the annotated genome of *B*. *cenocepacia* J2315 to identify putative c-di-GMP genes, which are assigned based on the presence of c-di-GMP metabolizing domains. The Burkholderia database (Winsor et al., 2008) was used to retrieve c-di-GMP related homologous genes from the genome of *B. cenocepacia* K56-2. In total, we identified 24 putative c-di-GMP-related genes in the genome of the K56-2 strain (Table 2). These genes encode GGDEF and/or EAL domain(s), but not HD-GYP domain, which is another c-di-GMP degrading domain. We then used the SMART bioinformatics software (Schultz et al., 2000; Letunic et al., 2015) to predict signaling (sensory or receiver) domains present in conjunction with the c-di-GMP metabolizing domains (Table 2). Twelve of the c-di-GMP genes encode sensory or receiver domains on their N-terminus in addition to the GGDEF and/or EAL domains (Figure 2A). Because we were interested in determining the role of signaling domain-containing c-di-GMP proteins in sensing nutritional cues present in CF sputum conditions and modulation of swimming motility, these 12 putative c-di-GMP metabolic proteins were chosen for further investigation. Among the sensory domains contained in the c-di-GMP proteins, the PAS and Cache domains are the most common (Table 2). Also, more than half of these proteins possess a transmembrane region, suggesting a periplasmic localization of some of these domains (Figure 2A). Among those, the only gene with an experimentally confirmed function in *B. cenocepacia* is *rpfR* (BCAM0580). RpfR is a PAS-GGDEF-EAL domain-containing protein that positively regulates swarming motility, protease activity, biofilm formation, and virulence (Deng et al., 2012). To find out whether the 12 sensory domain-containing c-di-GMP proteins mediate a change in intracellular c-di-GMP levels in response to CF sputum nutritional conditions, we created 12 c-di-GMP mutants using insertional mutagenesis and examined their swimming motility in SCFM. Except for BCAL1069, all other putative c-di-GMP synthesizing/degrading genes are not present in an operon. Therefore, a polar effect on downstream genes was not expected. All the c-di-GMP mutants showed growth kinetics similar to the WT strain in CF sputum nutritional conditions (Supplementary Figure 3). Among 12 c-di-GMP mutants, the WT::BCAM1161, WT::BCAM0580 (*rpfR*) and WT::BCAL1069 (*cdpA*) mutants exhibited a reduction in swimming motility in SCFM (Figure 2B). However, the WT::BCAL1069 (*cdpA*) mutant showed the most significant defect in swimming motility. The BCAL1069 protein is a homolog (86% identical) of CdpA that has been previously characterized in *B. pseudomallei* KHW (Lee et al., 2010). To rule out that phenotypes observed in WT::BCAL1069 (*cdpA*) could be caused by inactivation of the downstream gene BCAL1068, we created the insertional mutant WT::BCAL1068. The decrease in motility was not due to polar effects on BCAL1068 as the swimming motility of the WT::BCAL1068 mutant was similar to the WT strain (Supplementary Figure 4). To confirm that the reduced motility phenotype of WT::BCAL1069 (*cdpA*) mutant was due to the disruption of the BCAL1069 gene, we complemented the mutant with BCAL1069 (*cdpA*) *in trans*. The complemented strain showed partial restoration of the swimming motility, demonstrating that the defect in WT::BCAL1069 (*cdpA*) motility is due to the inactivation of the BCAL1069 (*cdpA*) gene (Figure 3A). In our previous study, we showed that SCFM conditions induced swimming motility through an increased flagellin protein expression (Kumar and Cardona, 2016). Then, we considered whether the mutation in BCAL1069 (*cdpA*) in the WT::BCAL1069 (*cdpA*) mutant affects flagellin expression and flagellation. Western blot and electron microscopy analysis demonstrated no difference in flagellin expression and flagella biosynthesis (Figures 3B,C, Supplementary Figure 6). Together, our results suggest that BCAL1069 (*cdpA*) positively regulates swimming motility of *B. cenocepacia* K56-2 in CF sputum nutritional conditions, without affecting flagellin expression.

**Table 2:** Putative c-di-GMP-related proteins in *B. cenocepacia* K56-2.

**Domains coded by c-di-GMP metabolizing proteins**	Number of genes encoding c-di-GMP proteins^*^
GGDEF only	4
EAL only	6
GGDEF and EAL	2
GGDEF and/or EAL with sensory domain (GAF, PAS, HAMP, Cache)	11
GGDEF with receiver domain (REC)	1

* *Represents total number c-di-GMP genes in the genome of B. cenocepacia K56-2*.

### BCAL1069 negatively regulates intracellular c-di-GMP levels in *B. cenocepacia* K56-2

**Figure 4:**
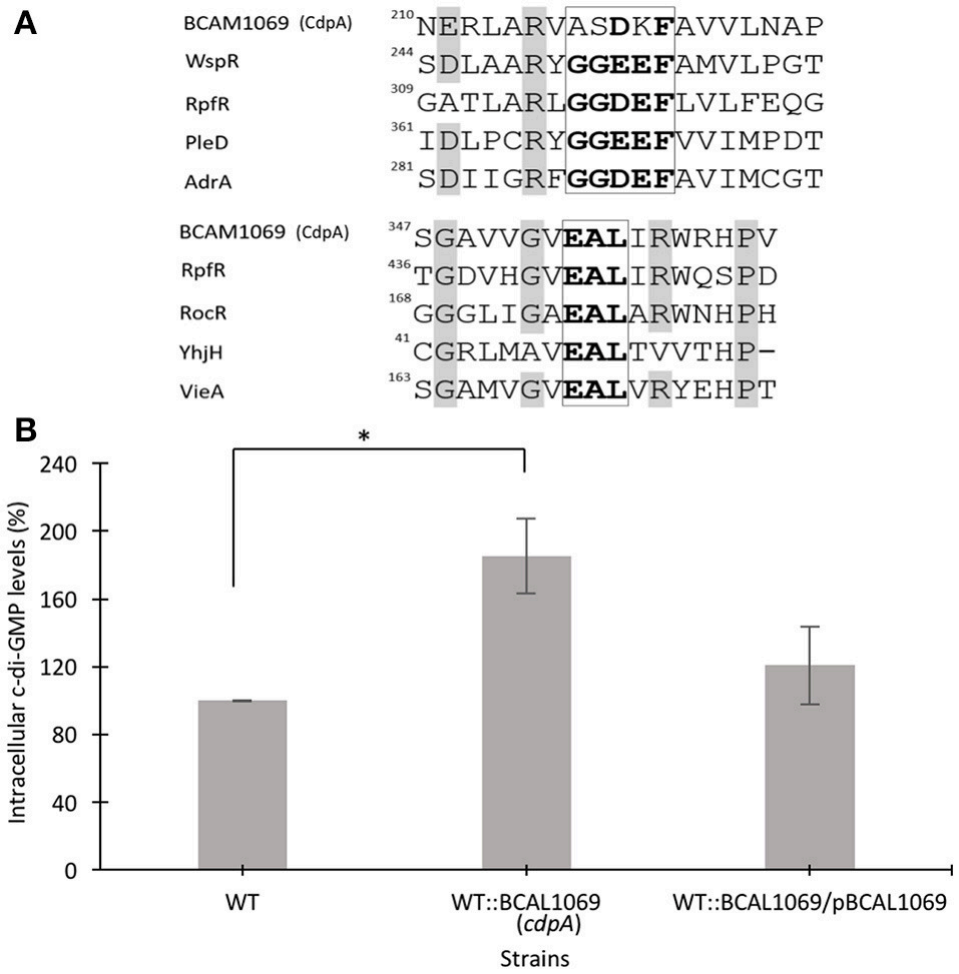
Multiple sequence alignment of residues of signature motifs of BCAL1069 (CdpA) and determination of intracellular levels of c-di-GMP in the BCAL1069 mutant. **(A)** Partial alignment of BCAL1069 amino acid sequence with GGDEF and EAL domain-containing proteins, retrieved from the UniProt database. The amino acids in the GGDEF and EAL motifs are highlighted in bold. Other conserved amino acids are shaded in gray. The numbers show the position of the amino acid in the protein. BCAL1069, UniProt Id B4ED05; WspR, UniProt Id Q9HXT9; RpfR, UniProt Id B4EKM4; PleD, UniProt Id Q9A515; AdrA, UniProt Id Q9L401; RocR, UniProt Id Q9HX69; YhjH, UniProt Id P37646; VieA, UniProt Id O68318. **(B)** Relative intracellular c-di-GMP levels in the WT:BCAL1069 mutant. Percentage represents area under the c-di-GMP curve of the strain relative to the WT strain. Three independent experiments were performed. ^*^Denotes significant *p*-values (*p* < 0.01).

Further analysis of the BCAL1069 (*cdpA*) protein sequence shows that the protein contains a PAS (*P*er/*A*rnt/*S*im) domain at the N-terminus, which is fused to the GGDEF and EAL domains. The protein has no predicted transmembrane-spanning regions, indicating a cytoplasmic location (Figure 2A). The PAS domain, which senses small molecule ligands, is widely present in sensory and signal transduction proteins in bacteria (Ulrich et al., 2005). A multiple sequence alignment of signature motifs (Katoh and Standley, 2013; Larsson, 2014) of the BCAL1069 protein (CdpA) and other GGDEF domain-containing proteins indicates that the GGDEF domain of BCAL1069 is an enzymatically inactive variant, since a degenerate GGDEF motif was observed (Figure 4A). In *Caulobacter crescentus*, a similar catalytically inactive variant of the GGDEF motif was detected in a c-di-GMP metabolizing protein, CC3396 (Christen et al., 2005). The presence of a PAS domain and an intact EAL motif in BCAL1069 suggests that this protein is likely a PDE in which the c-di-GMP degradation catalytic activity responds to an extracellular signal.

The presence of an inactive GGDEF and an active EAL domain in the BCAL1069 protein motivated us to investigate the intracellular c-di-GMP levels in the WT::BCAL11069 (*cdpA*) mutant. We extracted the nucleotide pools from the WT, WT::BCAL1069 (*cdpA*) and complemented WT::BCAL1069/pBCAL1069 strains grown in SCFM. The HPLC data showed that the insertional mutagenesis in BCAL1069 resulted in approximately two-fold increase in the intracellular c-di-GMP levels (Figure 4B). Moreover, *in trans* expression of the BCAL1069 gene in the mutant restored intracellular c-di-GMP levels similar to the WT strain, suggesting that the increased levels of c-di-GMP are caused by interruption of the BCAL1069 gene (Figure 4B). Taken together, the increased intracellular c-di-GMP levels of the WT::BCAL1069 mutant suggest that the BCAL1069 protein possesses PDE activity.

### BCAL1069 protein regulates swimming motility in response to arginine and glutamate amino acids

**Figure 5:**
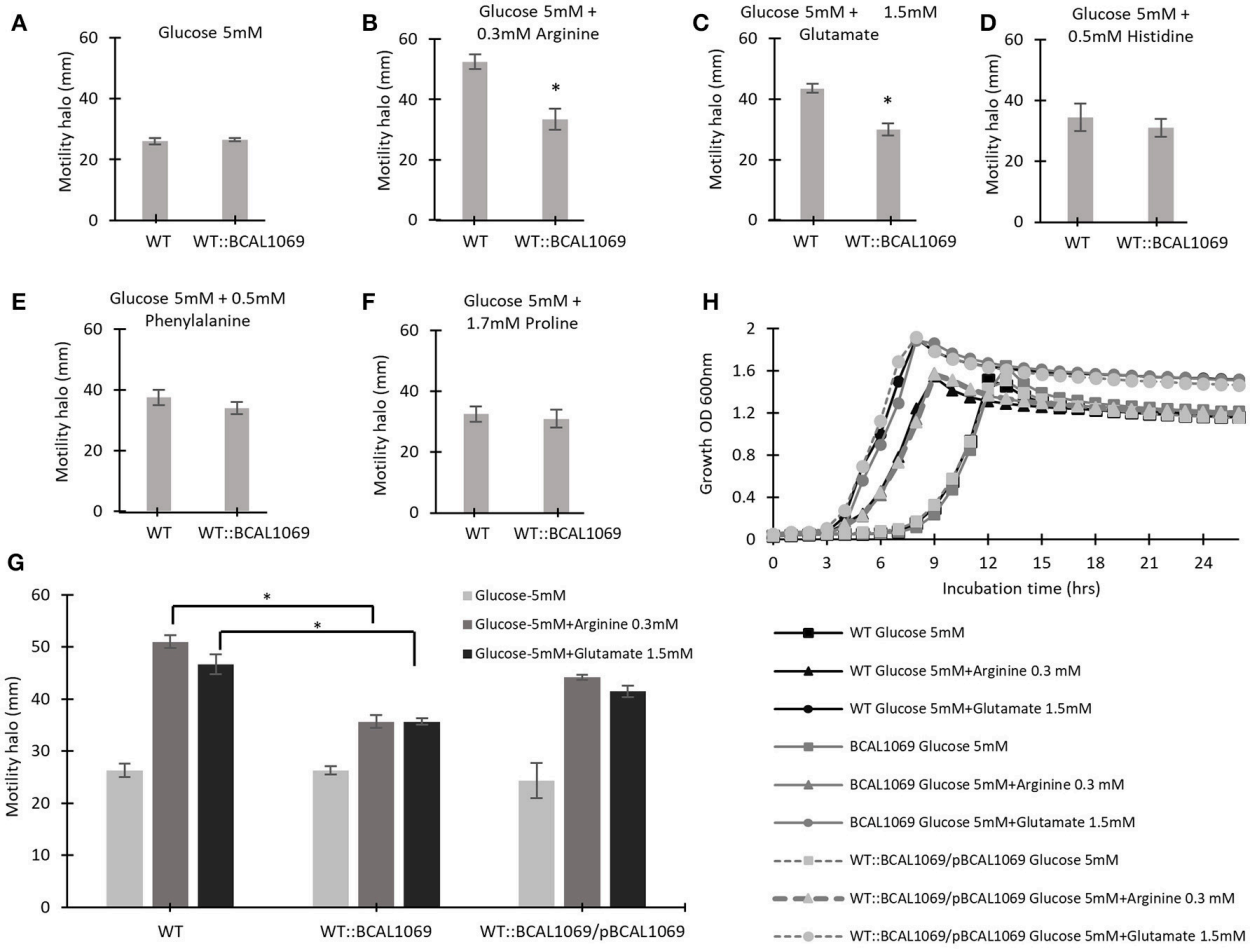
The effect of individual amino acids on the swimming motility of WT and WT::BCAL1069 mutant. **(A–G)** The motility halos of the indicated strains were examined in MOPS-glucose 5 mM with individual amino acids at the same concentration as present in SCFM, after incubation for 24 h at 37°C. Each experiment was performed three times independently in duplicate (^*^p < 0.01). **(H)** Growth curves of WT, WT::BCAL1069 and WT::BCAL1069/pBCAL1069 in MOPS-glucose 5 mM with individual amino acid at the same concentration as present in SCFM. The figure is representative of one from two independent experiments.

To identify if the BCAL1069 protein (CdpA) protein senses particular nutritional cues in CF sputum nutritional conditions, the swimming motility of the WT::BCAL1069 mutant was examined in glucose-minimal medium supplemented with individual amino acids at the same concentrations as found in SCFM. The amino acids, arginine, glutamate, histidine, phenylalanine, and proline, were chosen because the swimming motility of *B. cenocepacia* K56-2 was increased in the presence of those amino acids, compared with that of cells in the presence of glucose alone (Kumar and Cardona, 2016). The swimming motility of WT and WT::BCAL1069 was similar in the presence of glucose with or without histidine, phenylalanine, or proline (Figures 5A,D–F). However, the swimming motility of the BCAL1069 mutant was not influenced by arginine or glutamate as it was for the wild type (Figures 5A–C,G). Moreover, complementation with multicopy BCAL1069 restored the effect of arginine or glutamate to the mutant strain (Figure 5G). The lack of swimming motility changes of the WT::BCAL1069 mutant in response to arginine and glutamate was not due to impaired growth, since similar growth kinetics of the WT, WT::BCAL1069, and complemented mutant strains were observed in the medium (Figure 5H). Taken together, these results suggest that BCAL1069 is either directly or indirectly involved in sensing the presence of arginine and glutamate and induce swimming motility in *B. cenocepacia* K56-2.

### BCAL1069 protein regulates protease activity in *B. cenocepacia* K56-2

**Figure 6:**
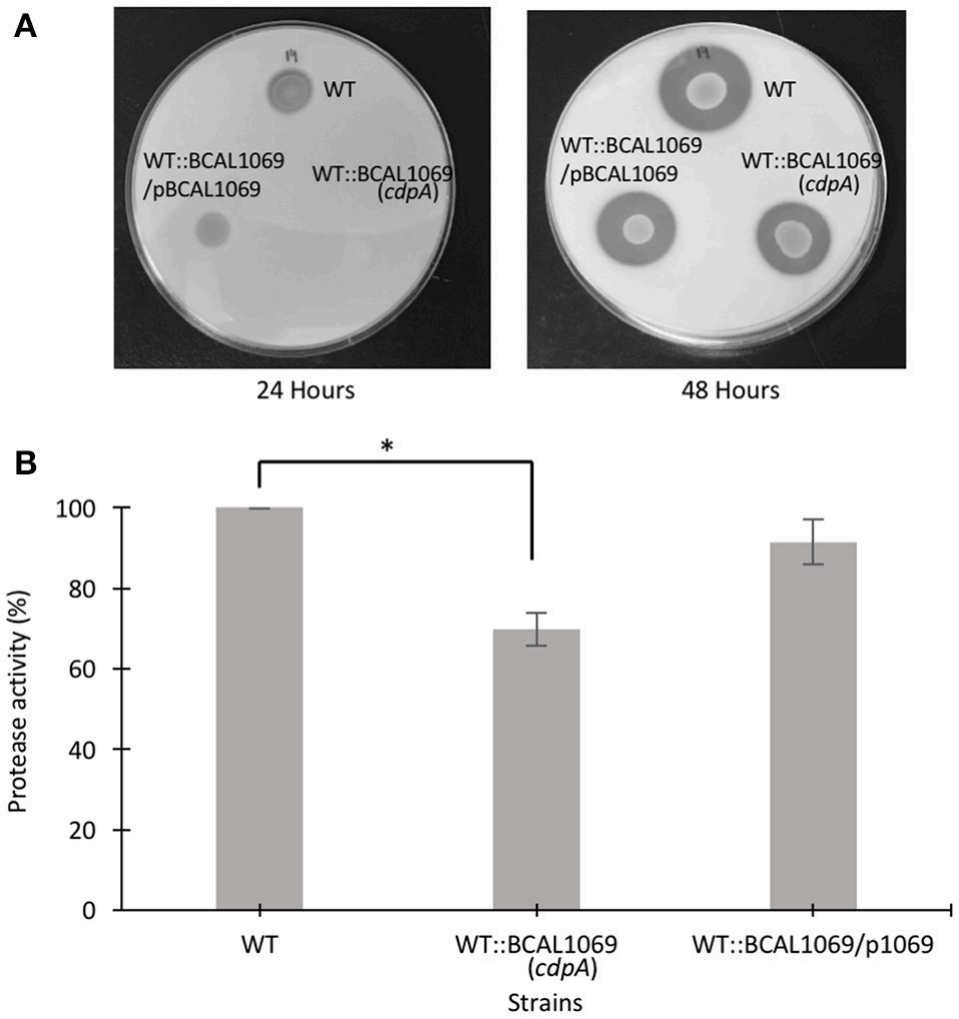
Protease activity of *B. cenocepacia* K56-2 WT, WT::BCAL1069 c-di-GMP mutant and complemented mutant. **(A)** Protease activity of strains on 2% skim milk agar plates after 24 and 48 h at 37°C. One representative of four independent experiments is shown. **(B)** Quantitative analysis of proteolytic activity after 48 h on 2% skim milk agar plates. Protease activity is represented in percentage relative to the WT strain. The protease assay was performed four times independently with three technical replicates per experiment (^*^p < 0.01).

Protease activity and biofilm formation have been associated with c-di-GMP regulatory networks (Deng et al., 2012; Ryan et al., 2015). To study the functional relationship between the BCAL1069 (CdpA) protein and virulence factors, we investigated whether BCAL1069 regulates protease activity and biofilm formation in *B. cenocepacia* K56-2. To examine protease activity, a clear zone around inoculated bacterial spots in the skim milk plates was measured at two different time points. After 24 h, low level of protease activity was observed for WT and complemented strains. To quantify the protease activity of the mutants, clear zones were observed for the strains after 48 h of incubation (Figure 6A). The WT::BCAL1069 (*cdpA*) mutant showed a significant reduction in protease activity in comparison to the WT strain (Figure 6B). In the complemented WT::BCAL1069/pBCAL1069 strain, the defect in protease activity was restored to WT levels, suggesting the reduced protease activity is caused by insertional mutagenesis of BCAL1069 (Figure 6B). When the biofilm formation ability of the WT::BCAL1069 (*cdpA*) mutant was analyzed in comparison to the WT strain, there was no significant difference observed (Supplementary Figure 5). Taken together, the BCAL1069 regulates protease activity, but not biofilm formation in *B. cenocepacia* K56-2.

## Discussion

C-di-GMP, a second messenger intracellular signaling molecule, is involved in the regulation of a wide range of bacterial processes and its metabolizing proteins are part of signal transduction pathways (Römling et al., 2013). The intracellular levels of this molecule are modulated by GGDEF or EAL domain-containing c-di-GMP proteins. The c-di-GMP metabolizing domains, GGDEF, and/or EAL, have been identified in conjunction with signaling (sensory and receiver) domains. Sensory and receiver domains are an integral part of signal transduction pathways and mechanisms (Galperin et al., 2001). For instance, in *P. aeruginosa*, WspR and RocR c-di-GMP proteins are comprised of a receiver (REC) in addition to the GGDEF or EAL domain (Hickman et al., 2005; Rao et al., 2008). On the contrary, the STM1987 protein of *S. Typhimurium* possesses sensory Cache1 and GGDEF domains and is involved in cellulose synthesis associated with biofilm formation (Mills et al., 2015). Other sensory domains are also linked to the c-di-GMP metabolizing domains, such as HK, PAS, GAF, HAMP, and REC (Galperin et al., 2001; Galperin, 2006).

Studies have shown that c-di-GMP plays a role in swimming and swarming motility, protease activity, biofilm formation, and virulence in several bacteria (Deng et al., 2012; Wei et al., 2016; Plumley et al., 2017). For instance, *P. aeruginosa* induces c-di-GMP levels through two c-di-GMP genes, s*adC* and s*iaD*, to upregulate biofilm formation and counter stress when exposed to tellurite (Chua et al., 2015). A systematic analysis of 11 GGDEF, EAL, and signaling domain-containing proteins identified two c-di-GMP mutants (*XOC_2393* and *XOC_4190*), which positively modulated swimming motility and virulence in the rice pathogen, *X. oryzae* (Wei et al., 2016). In *B. pseudomallei*, two PDE, I2284::T24 (*cdpA*) and I2285::T24, encoding genes positively modulate swimming motility irrespective of temperature (30°C and 37°C), whereas, a diguanylate cyclase mutant, II2523::T24, regulates biofilm formation in a temperature dependent manner (Plumley et al., 2017). Moreover, CdpA regulated flagellum biosynthesis, motility, biofilm formation, aggregation, and cell invasion (Lee et al., 2010). Despite the established role of c-di-GMP genes in motility and the presence of 24 c-di-GMP genes in *Burkholderia* genomes, only one c-di-GMP gene, *rpfR* (BCAM0580), has been characterized in *B*. *cenocepacia* previously to this study. This gene encodes PAS-GGDEF-EAL domains. The interaction of *Burkholderia* diffusible signal factor with the PAS domain of RpfR resulted in decreased intracellular c-di-GMP levels, which consequently regulated swarming motility, biofilm formation, and protease activity (Deng et al., 2012). However, the genome of *B. cenocepacia* K56-2 encodes 12 c-di-GMP proteins that also code for sensory domain, including the RpfR (BCAM0580) protein (Table 2 and Figure 2A). The identification of sensory domains in c-di-GMP-related proteins in *B. cenocepacia* indicates the presence of signal transduction pathways that may sense external signals to control swimming motility through a c-di-GMP turnover.

In the present study, we created 12 c-di-GMP insertional mutants and examined their swimming motility in CF sputum nutritional conditions. Three c-di-GMP mutants, WT::BCAM0580 (*rpfR*), WT::BCAM1161, and WT::BCAL1069, demonstrated a reduction in the swimming motility phenotype (Figure 2B). We chose the WT::BCAL1069 mutant, a homolog of the *cdpA* gene in *B. pseudomallei* KHW, for further studies, since the WT::BCAL1069 mutant displayed the most significant swimming motility defect (Figure 2B). Like *rpfR*, the BCAL069 (*cdpA*) gene is involved in the regulation of protease activity; however, BCAL1069 is not associated to biofilm formation in *B. cenocepacia* under the conditions tested (Figure 6 and Supplementary Figure 5). Unlike the BCAL1069 protein (CdpA) in *B. cenocepacia* K56-2, the *B. pseudomallei* KHW CdpA positively modulated biofilm formation (Lee et al., 2010). Moreover, the BCAL1069 mutant showed an increase in intracellular c-di-GMP levels, which could be explained by the presence of an active EAL domain and a degenerate GGDEF domain (Figure 4). The presence of a sensory PAS domain in the BCAL1069 protein (CdpA; Figure 2A), suggested that signal molecules may act to induce conformational changes to its linked enzymatic domain (Ulrich et al., 2005; Deng et al., 2012). We then hypothesized that the BCAL1069 protein responds to specific nutritional cues in CF sputum conditions, namely amino acids. To investigate if the PAS domain-containing BCAL1069 protein senses any specific amino acids in CF sputum conditions, swimming motility of the WT::BCAL1069 mutant was examined in glucose-minimal medium supplemented with individual amino acids. Our results suggested that swimming motility of the WT::BCAL1069 mutant was not influenced in the presence of arginine and glutamate (Figure 5). These results indicate that BCAL1069 (CdpA) has a role in mediating *B. cenocepacia* swimming motility by sensing the amino acids arginine and glutamate. It would be useful to further demonstrate if the PAS domain of BCAL1069 (CdpA) protein directly senses amino acids, arginine and glutamate. Other c-di-GMP proteins have been shown to directly or indirectly sense amino acids to modulate intracellular c-di-GMP levels, transducing the signal into an increase in cellulose synthesis (Mills et al., 2015) and biofilm dispersion (Basu Roy and Sauer, 2014).

C-di-GMP regulatory networks are comprised of signal transduction systems, where c-di-GMP regulate a biological process by binding to diverse c-di-GMP receptors at multiple steps (Orr et al., 2016). Once c-di-GMP is bound to its receptors, the resulting complex controls and affects bacterial phenotype(s) or cellular processes at the level of transcription, translation or post translation (Shanahan and Strobel, 2012). Swimming motility is one of the bacterial phenotypes regulated by c-di-GMP at the transcriptional and post-translational level under elevated c-di-GMP levels. For instance, in *P. aeruginosa*, the FleQ master regulator downregulates expression of flagellar genes (Hickman and Harwood, 2008; Baraquet and Harwood, 2013). Whereas, in *E. coli*, the interaction of c-di-GMP with flagella motors and YcgR proteins control functioning of flagella (Fang and Gomelsky, 2010; Paul et al., 2010). We demonstrated that the high intracellular c-di-GMP levels in the WT/pWspR strain reduce swimming motility without affecting the flagellin expression and flagellar biosynthesis, suggesting a post translational regulation of swimming motility in *B. cenocepacia* K56-2 (Figure 1 and Supplementary Figure 2). Further, our results showed that the swimming motility of WT::BCAL1069 (*cdpA*) mutant was reduced without changing the flagellin expression and flagellation, while the mutant displayed increased intracellular levels of c-di-GMP (Figures 3, 4). This indicates that c-di-GMP regulates swimming motility by induced intracellular c-di-GMP levels independent of flagellin expression. Noticeably, in *B. pseudomallei* KHW, the *cdpA* mutant exhibited aflagellate phenotype (Lee et al., 2010), which is in contrast to the WT:: BCAL1069 (*cdpA*) mutant flagellate phenotype observed in this study (Figure 3). This difference in swimming motility at the level of flagella biosynthesis in the two *cdpA* mutants suggests that c-di-GMP signaling transduction pathways can regulate motility through gene expression, assembly of flagellar proteins or flagellar functioning despite belonging to two species of the same genus. Bacteria residing in diverse ecological niches develop more complex signaling systems than those that dwell in stable environmental settings (Galperin, 2005). *Burkholderia* species have been isolated from diverse ecological niches, such as soil, water bodies, rhizosphere, and lungs of CF patients (Mahenthiralingam et al., 2005). When *B. cenocepacia* encounters the host environment, there must be mechanisms in place that allow the cells to sense the lung nutritional conditions, which is rich in amino acids. Once *B. cenocepacia* establishes an infection in the lungs of CF patients, bacteria may undergo distinct adaptive strategies, including mutations in motility related regulators (Lee et al., 2017). These late genetic mutations indicate that *B. cenocepacia* can adapt to the CF lungs and loss motility phenotype during chronic infection to evade host immune response. The host Toll-like receptor 5 recognizes flagellin as a ligand to induce the inflammatory defense response to eradicate pathogens (Hayashi et al., 2001). Previously, we demonstrated that the CF sputum conditions induce swimming motility, flagellin expression and multiple flagellation through the *flhF* gene in *B. cenocepacia* K56 (Kumar and Cardona, 2016). In this study, we unravel another link between *B. cenocepacia* swimming motility and nutritional cues in CF conditions, by establishing the integration of the second signal messenger c-di-GMP into the swimming motility response to amino acids. We propose that sensing of nutritional cues in the CF lung, followed by an increase in c-di-GMP-mediated swimming motility, plays an important role in the establishment of the initial stage of *B. cenocepacia* infection. It remains to be determined if the other c-di-GMP genes, not investigated in this study, also have a role in virulence and swimming motility.

## Author contributions

BK: Designed and performed the experiments, interpreted the data, and wrote the manuscript; JS: Overviewed the HPLC experiments and edited the final version of the manuscript; SC: Conceived the research approach, contributed to writing, and edited the final version of the manuscript.

### Conflict of interest statement

The authors declare that the research was conducted in the absence of any commercial or financial relationships that could be construed as a potential conflict of interest.
