# Supplementary material for: A c-di-GMP-Modulating Protein Regulates Swimming Motility of Burkholderia cenocepacia in Response to Arginine and Glutamate
Source: Front Cell Infect Microbiol. 2018 Feb 28;8:56. doi: 10.3389/fcimb.2018.00056 (PMC5835511; doi:10.3389/fcimb.2018.00056)
Supplement: Supplementary file 1 [file Table1.DOCX]

**Supplementary Table 1. Primers used in this study**

| **No.** | **Primer name** | **Sequence** |
| --- | --- | --- |
| 686 | PAO1 *wspR* R | ATTACATATGCACAACCCTCATGAGAGCAAG |
| 687 | PAO1 *wspR* R | ATATTCTAGATACTGCACTTGCGCCCCACAG |
| 782 | WT::BCAM1160 F | ATTATCTAGAAAAGTTCTCGCTACAGCATCGG |
| 783 | WT::BCAM1160 R | ATTAGAATTCATCGGTTTCGATGTCCTCG |
| 784 | WT::BCAM1554 F | ATATTCTAGATCGATGCTCGTCACGCTGTTCG |
| 785 | WT::BCAM1554 R | ATTAGAATTCGCATCGCCGCGAGATAGATC |
| 786 | WT::BCAM2836 F | ATATTCTAGATCGCTGAGCGACCTCGTGATC |
| 787 | WT::BCAM2836 R | ATTAGAATTCAGCGGCATGAACGTGCAGTC |
| 788 | WT::BCAL1020 F | TAATTCTAGATGCTACAGCTATTCCAAAGCCG |
| 789 | WT::BCAL1020 R | ATATGAATTCTGACGTAAAGCCCCACGTTCG |
| 790 | WT::BCAM1161 F | ATTATCTAGAATGGTGCTGCTGGTCGACG |
| 791 | WT::BCAM1161 R | ATATGAATTCGCAGCAGGTTCAGGTACGAG |
| 792 | WT::BCAL1069 F | ATTATCTAGAGCAACTGGCTGGAACAGACG |
| 793 | WT::BCAL1069 R | ATATGAATTCGTCGTGGTGCATTTCGTAC |
| 794 | WT::BCAM0580 F | TATATCTAGAACCTCGTGCTCGACATCTTGC |
| 795 | WT::BCAM0580 R | ATTAGGTACCCGAAAAAGCCCGTGATGTTGC |
| 832 | WT::BCAL2852 F | ATTATCTAGACCTGTGCGTGCTACAGCTAT |
| 833 | WT::BCAL2852 R | ATTAGAATTCGGTGGATCGTGAAGTATCTG |
| 834 | WT::BCAM0748 F | TAATTCTAGATCGTGGCACGCATTCTGTCG |
| 835 | WT::BCAM0748 R | TAATGAATTCCACGAAAATGGAGCCGAGATAGG |
| 836 | WT::BCAM2822 F | ATATTCTAGATTCCTGCTGCCGTACCTGATC |
| 837 | WT::BCAM2822 R | TAATGAATTCTAGATGCCGCCGAGGTACTTG |
| 866 | WT::BCAM2256 F | ATTATCTAGAGCAACAACCTGAACCTGCTG |
| 867 | WT::BCAM2256 R | TATAGAATTCCGAGCAGGAATCCGAAGAAC |
| 869 | WT::BCAM1670 F | TATATCTAGAATCGTGGTCAATCACGAGACG |
| 870 | WT::BCAM1670 R | ATTAGAATTCATTGAGCATCACGTTCATGCG |
| 857 | WT::BCAL1068 F | ATTATCTAGACGATGAAGAACGACGACCTCG |
| 858 | WT::BCAL1068 R | ATTAGAATTCATCAGCGTGCAGACGACGG |
| 461 | P53 pGpΩTp | TAACGGTTGTGGACAACAAGCCAGGG |
| 825 | WT::BCAM1160 Up | GGGCACGAAAAGGCACGAAATATCG |
| 826 | WT::BCAM1554 Up | TTCCGTTCGCAACGATGCTGCCT |
| 827 | WT::BCAM2836 Up | CATTTCGTGATGCTGATGGCCGAG |
| 828 | WT::BCAL1020 Up | CCCTTATTTCGTTGTGGCAGTCGG |
| 829 | WT::BCAM1160 Up | GCATCGACCATGACGATTGACCTGAC |
| 830 | WT::BCAL1069 Up | CTTCACGTCAGCGAGAAACATGGAAGC |
| 838 | WT::BCAL2852 Up | GAGACTTTGTATGCGAAATCACGTCCGACC |
| 839 | WT::BCAM0748 Up | CGGCAATACGCAAAATCGACCGATC |
| 840 | WT::BCAM2822 Up | GGTAGTCATACGAACAATCGCATCGACC |
| 868 | WT::BCAM2256 Up | TCGACATCAATCTCGATGCCGTCG |
| 871 | WT::BCAM1670 Up | CACGAGCATCTCGTATGGCTTCAGC |
| 859 | WT::BCAL1068 Up | ACGGAGATGGGCTGCAATCACATCC |
| 876 | pKD3-CAT F | TGATGATATCTCATCGCAGTACTGTTGTATTC |
| 877 | pKD3-CAT R | \| TATAATGCATAAGTATAGGAACTTCGGCGC \| \| --- \| \|  \| |
| 851 | Complement BCAL1069 gene F | GAGACATATGGAAGCCAACAGGAAACAG |
| 852 | Complement BCAL1069 gene R | \| AGAGTCTAGATGGTTGACATACGGATCTGTC \| \| --- \| \|  \| |

* Bases underlined represent restriction enzyme recognition sites and respective name are given below:

CATATG - NdeI ; TCTAGA - XbaI ; GAATCC - EcoRI ; GGTACC – KpnI ; GATATC - EcoRV ; ATGCAT – NsiI
