# Supplementary material for: A c-di-GMP-Modulating Protein Regulates Swimming Motility of Burkholderia cenocepacia in Response to Arginine and Glutamate
Source: Front Cell Infect Microbiol. 2018 Feb 28;8:56. doi: 10.3389/fcimb.2018.00056 (PMC5835511; doi:10.3389/fcimb.2018.00056)
Supplement: Supplementary file 2 [file Image1.PDF]

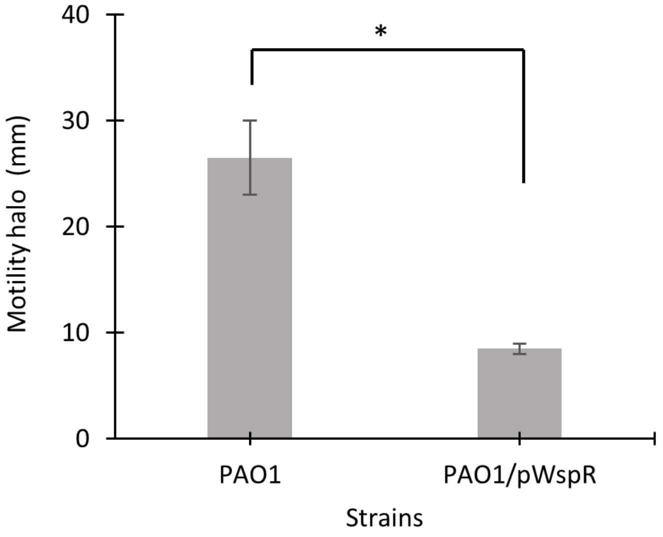

Supplementary Figure 1. Swimming motility of *P. aeruginosa* PAO1 in c-di-GMP modulating conditions. The bar graph shows swimming motility halos of strains with varying intracellular c-di-GMP levels. Motility of the strains was examined in LB semi-solid (0.3%) agar plates after 24 hours of incubation at 37°C. The motility assay was performed three-times independently in duplicates and '*' denotes significant p-values ($p<0.01$).
