# Supplementary material for: A c-di-GMP-Modulating Protein Regulates Swimming Motility of Burkholderia cenocepacia in Response to Arginine and Glutamate
Source: Front Cell Infect Microbiol. 2018 Feb 28;8:56. doi: 10.3389/fcimb.2018.00056 (PMC5835511; doi:10.3389/fcimb.2018.00056)
Supplement: Supplementary file 3 [file Image2.PDF]

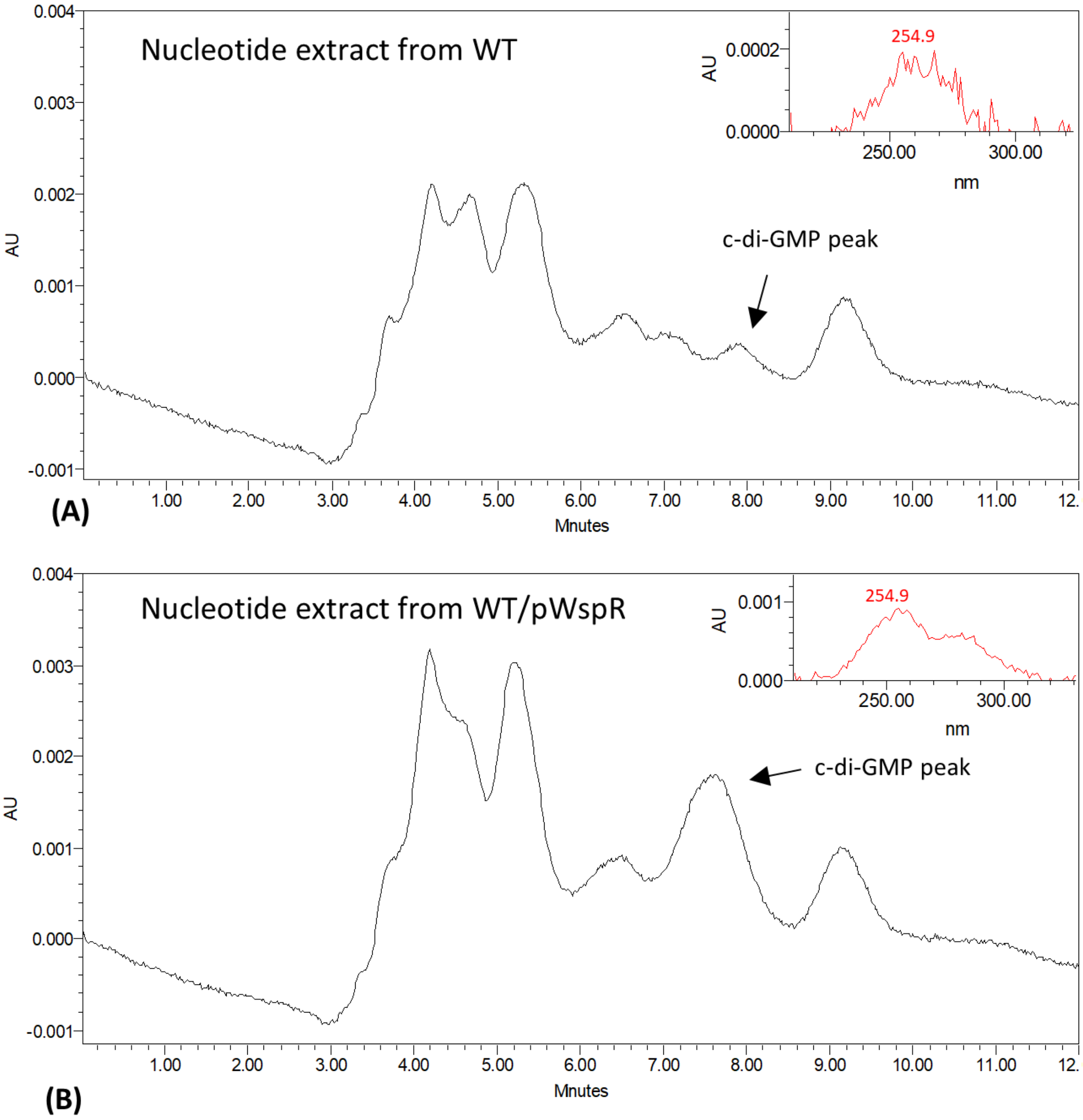

Nucleotide extract from WT
AU
0.004
0.003
0.002
0.001
0.000
-0.001
1.00
2.00
3.00
4.00
5.00
6.00
7.00
8.00
9.00
10.00
11.00
12.
Mnutes
c-di-GMP peak
254.9
0.0002
0.0000
250.00
300.00
nm
(A)
Nucleotide extract from WT/pWspR
c-di-GMP peak
254.9
0.001
0.000
(B)

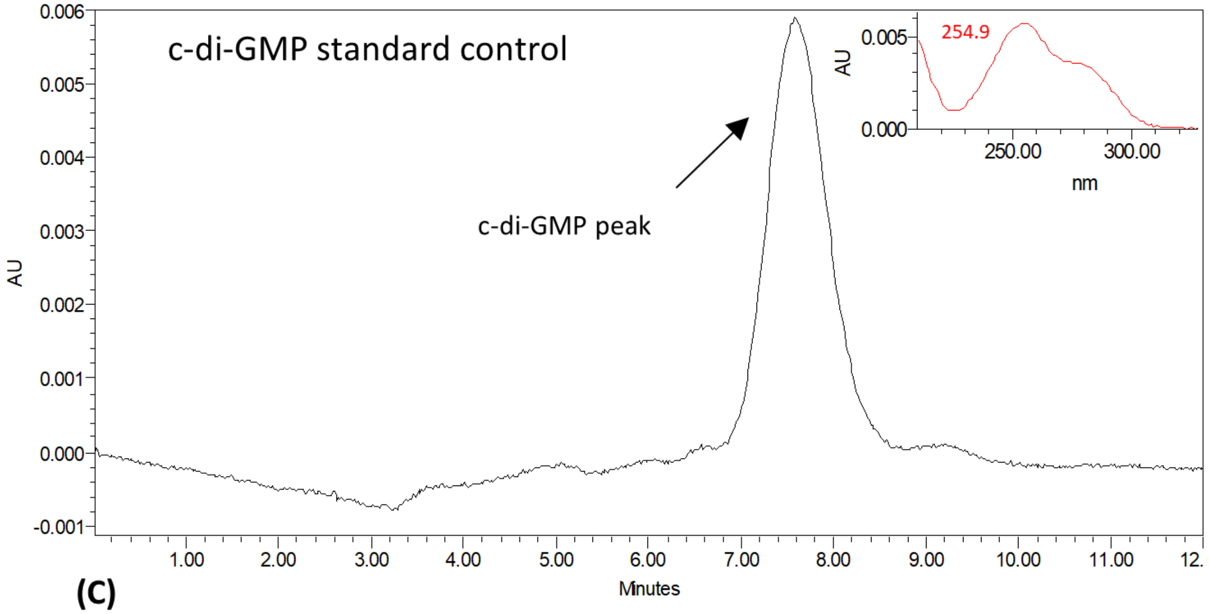

(C)

Supplementary Figure 2. Detection of intracellular c-di-GMP levels in *B. cenocepacia* K56-2 using HPLC. The peak of intracellular c-di-GMP is highlighted in the chromatograms of nucleotide extracts from WT **(A)**, WT/pWspR **(B)**. The retention time of c-di-GMP was approx. 7.6 min, which is identified on the basis of retention time of standard c-di-GMP and UV trace **(C)**.
