# Supplementary material for: A c-di-GMP-Modulating Protein Regulates Swimming Motility of Burkholderia cenocepacia in Response to Arginine and Glutamate
Source: Front Cell Infect Microbiol. 2018 Feb 28;8:56. doi: 10.3389/fcimb.2018.00056 (PMC5835511; doi:10.3389/fcimb.2018.00056)
Supplement: Supplementary file 4 [file Image3.PDF]

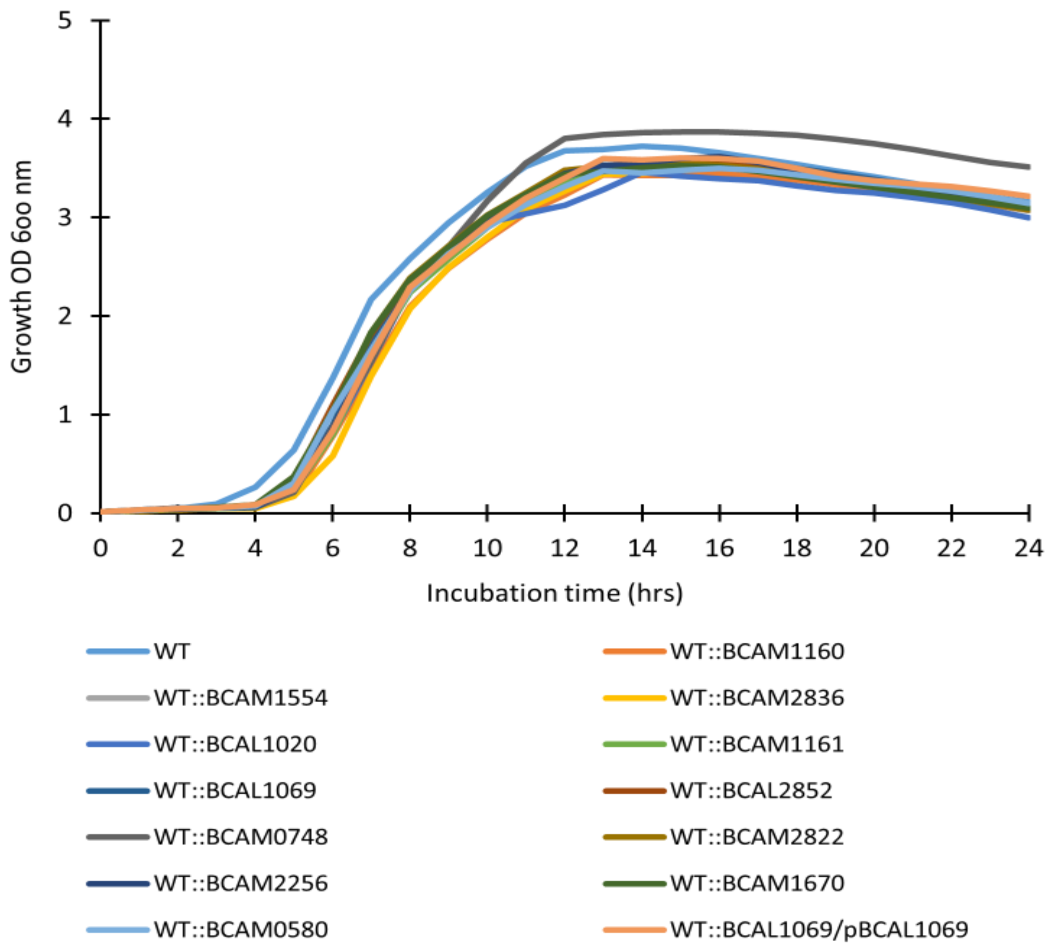

Supplementary Figure 3. Growth kinetics of *B. cenocepacia* K56-2 WT and twelve c-di-GMP mutants. The graph shows growth of the WT strain, twelve c-di-GMP mutants and complement WT::BCAL1069/pBCAL1069 in CF sputum conditions. All the strains were grown at 37 °C for 24 hours. The growth assay was performed twice and one representative is shown here.
