# Supplementary material for: A c-di-GMP-Modulating Protein Regulates Swimming Motility of Burkholderia cenocepacia in Response to Arginine and Glutamate
Source: Front Cell Infect Microbiol. 2018 Feb 28;8:56. doi: 10.3389/fcimb.2018.00056 (PMC5835511; doi:10.3389/fcimb.2018.00056)
Supplement: Supplementary file 5 [file Image4.PDF]

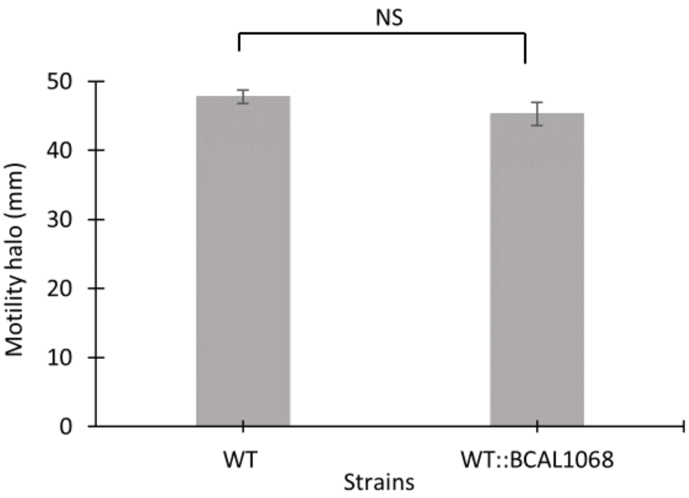

Supplementary Figure 4. Swimming motility of WT::BCAL1068 mutant. The bar graph shows swimming motility halos of the WT, WT::BCAL1068 mutants. Motility was measured in semi-solid 0.3% agar CF sputum conditions plates. The plates were incubated for 24 hours at 37°C. The motility assay was performed three-times independently in duplicates and 'NS' denotes no significance difference.
