# Supplementary material for: A c-di-GMP-Modulating Protein Regulates Swimming Motility of Burkholderia cenocepacia in Response to Arginine and Glutamate
Source: Front Cell Infect Microbiol. 2018 Feb 28;8:56. doi: 10.3389/fcimb.2018.00056 (PMC5835511; doi:10.3389/fcimb.2018.00056)
Supplement: Supplementary file 6 [file Image5.PDF]

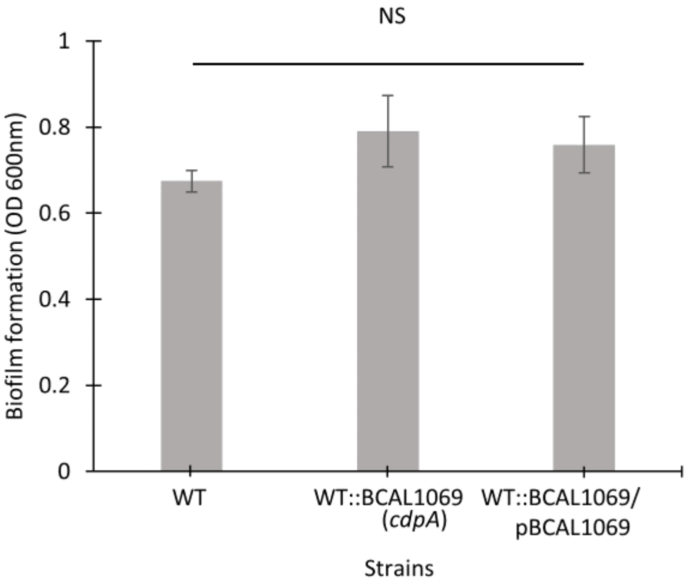

Supplementary Figure 5. Biofilm formation of *B. cenocepacia* K56-2 WT, WT::BCAL1069 c-di-GMP mutant and complemented mutant. The graph shows biofilm formation of the strains in 96 well plates after 48 hours of incubation time. The CV stained attached cells were resuspended in acetone: ethanol (20:80) and measured at OD_{600}. The biofilm assay was performed three times independently with four technical replicates. 'NS' represent no significant difference.
