# Supplementary figures and images for: A c-di-GMP-Modulating Protein Regulates Swimming Motility of Burkholderia cenocepacia in Response to Arginine and Glutamate
Source: Front Cell Infect Microbiol. 2018 Feb 28;8:56. doi: 10.3389/fcimb.2018.00056 (PMC5835511; doi:10.3389/fcimb.2018.00056)

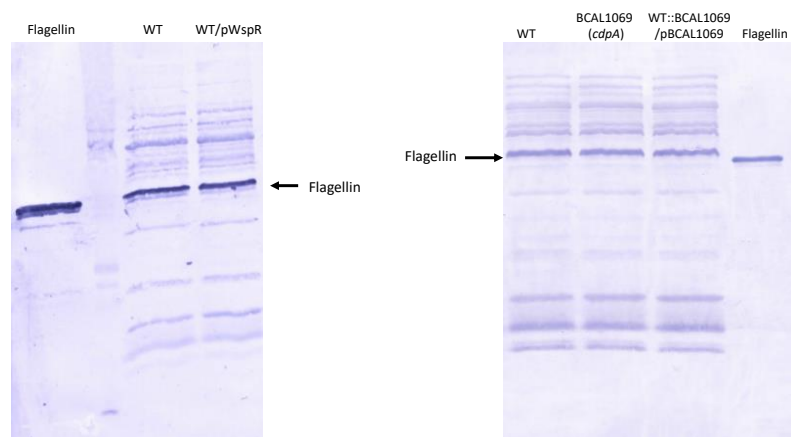

**Supplementary Figure 6. Western Blots corresponding to Figure 1B (left panel) and 3B (right panel)**

Supplement: Supplementary file 7 [file Image6.PDF]
